# Research on the influence of family capital on academic achievement of first-generation college students in China

**DOI:** 10.3389/fpsyg.2023.1174345

**Published:** 2023-08-24

**Authors:** Ying Zhao, Zhi Wang, Zengyuan Ren

**Affiliations:** ^1^The Tourism College of Changchun University, Changchun, China; ^2^School of Education, Liaoning Normal University, Dalian, China; ^3^Institution of Higher Education, Jilin University, Changchun, China

**Keywords:** family capital, academic achievement, ability development, academic performance, self-concept, structural equation model, first-generation college student

## Abstract

**Objective:**

The objective of this study is to explore the influence of family capital (including family economic capital, family cultural capital and family social capital) on the academic achievement (including ability development, academic performance and self-concept) of first-generation college students.

**Methods:**

The questionnaires are based on the CFPS (China Family Panel Studies) database and tailored to the specific circumstances. Data was collected from 1524 first-generation college students from five universities in Liaoning Province. SPSS 23.0 and AMOS 24.0 were used to analyze the data.

**Results:**

Family economic capital significantly predicted ability development (standardized regression coefficient = 0.198, *P* < 0.001) and academic performance (standardized regression coefficient = 0.220, *P* < 0.001); Family cultural capital significantly predicted ability development (standardized regression coefficient = .114, *P* < 0.001), academic performance (standardized regression coefficient = 0.217, *P* < 0.001) and self-concept (standardized regression coefficient = 0.160, *P* < 0.001); Family social capital significantly predicted academic performance (standardized regression coefficient=0.084, *P* < 0.01) and self-concept (standardized regression coefficient = 0.156, *P* < 0.001).

**Conclusion:**

Family capital can significantly affect the academic achievement of first-generation college students. To bridge the gap of academic achievement caused by family capital for first-generation college students and promote class mobility, special attention should be paid to the internal actions of students in higher education fields, the connection between different fields should be strengthened, and humanistic care for disadvantaged groups should be implemented.

## 1. Introduction

Against the backdrop of accelerated popularization of higher education and favorable national policies, more and more students from ordinary families have the access to university. At the same time, the structure of the student group also shows diversity and differences. Relevant survey also points out that not less than 70% of college students in China’s undergraduate colleges and universities are “first-generation college students in families” (Zhang et al., 2017), which refers to full-time college students whose parents have not received tertiary education at junior college level or above and this definition does not involve the education levels of grandparents or siblings of these college students in the family. As higher education enters a new stage of universalization, first-generation college students have more equitable access to higher education, but still face many problems and challenges in the process of education. The success brought by college entrance exams may seem to open the door to social mobility, but it does not necessarily mean that they can adapt to college life and obtain the same academic achievement and social experience as non-first-generation college students after entering campus. When many scholars study these influences, they first consider the ascribed factors, for example, family background. Related studies have shown that family capital has a significant impact on college students’ academic achievement, which means that differences in family capital significantly affect students’ academic achievement and if such differences continue to expand, it will inevitably affect the children of the lower class to achieve social mobility.

As a major group of students, first-generation college students have all the necessity of the research. By investigating the group characteristics of the first-generation college students in families, we can analyze more accurately which factors in the family capital affect the academic achievement of first-generation college students in families, and help students to take timely measures to bridge their inherent gaps according to their actual situation. It is of great value and significance to figure out the factors influencing the academic achievement of first-generation college students. For families, a more reasonable investment will be put in their education for better academic achievements so as to avoid the waste of educational resources; as for schools, different measures can be taken to show more care for vulnerable groups and then ensure the fairness of education. At the same time, it is worth reflecting on whether higher education and the university field, as links for students’ development, perpetuate class stratification or achieve class mobility? Through the analysis of the influences of family capital on the academic achievement of first-generation college students, this paper attempts to answer the influences mechanism of family capital, hoping to provide more ideas for the research on the influence related to the academic achievement of first-generation college students.

## 2. Literature review

### 2.1. Research on the connotation of family capital

The term “family capital” originated from the theory of social capital. Coleman et al. (1966) pointed out that family capital is a part of social capital and covers the occupation, social status, economic conditions and education level of parents, etc., which is owned by individuals, expounded in interpersonal relations, and provides convenience for individual actions. In *Forms of Capital*, Bourdieu (1986) pointed out that the three basic forms of capital are economic capital, cultural capital and social capital, and these three kinds of capital are not independent of each other. According to Bourdieu’s “exchange principle,” the three kinds of capital can be transformed into each other under certain conditions (Bourdieu, 1986). Coleman (1988) believed that there are three forms of capital, namely, social capital, physical capital, and human capital. Among them, social capital is a capital property characterized by social structure resources owned by individuals, which is provided by family, neighborhood, community and other primitive social organizations, and is divided into social capital within the family and social capital outside the family. The forms of social capital are obligations and expectations, social information networks, norms and effective punishments, authority relations and social organizations. In Coleman’s opinion, the connotation of family capital is relatively broad, and significantly affects students’ academic achievement.

In China, research on family capital is based on the capital theory of Bourdieu and Coleman, and expands related to China’s social environment. Liu and Gao (2011) interpreted family capital as the family background that college students use to achieve some “instrumental purposes,” which is divided into four dimensions: family economic capital, family cultural capital, family political capital and family social capital. Family economic capital is represented by family annual income and inherent assets, and family cultural capital is represented by the cultural level of the father. The political status of the family and the administrative rank of the post are used to represent the political capital of the family, and the father’s occupation, family social status and the breadth of human connections are used to measure the family social capital. Jiang and Yan (2006) divided family capital into three dimensions: family economic capital, family cultural capital and family social capital. Family income, family assets and father’s occupation reflected family economic capital stock; father’s education level, family school supplies and collection reflected family cultural capital stock; and connection between parents and children’s learning reflected family social capital.

Based on previous research by scholars on family capital and the division of its dimensions, this paper defines family capital as the sum of family members’ economic wealth, parents’ occupation, parents’ cultural level and the stock of social interpersonal relationship. And in this paper, family capital is divided into three dimensions: family economic capital, family cultural capital, and family social capital.

### 2.2. Influencing factors of academic achievement

Most scholars study the influencing factors of academic achievement from three aspects: individual, school and family. For example, Wang and Liu (2000) pointed out that intelligence level can be used to explain the difference in students’ academic achievement, which is fully demonstrated in the difference in the way of students’ thinking. In addition, some scholars have found that there is a significant relationship between students’ academic achievement and individual psychological factors, among which, goal awareness and professional emotions have a positive impact on college students’ academic achievement, while learning process pressure has a negative impact on college students’ academic achievement. Other studies have shown that gender factors will also have an important impact on college students’ major learning. Male students take the dominant position in both high and low groups, and the gender differences in academic performance will continue to deepen in the upper grades. Students can make full use of their abilities and advantages by adjusting their learning state and developing learning methods suitable for themselves, so as to achieve pre-set learning goals and achieve satisfactory academic achievements.

Zhao et al. (2020), in studying the influence of school environment on college students’ academic achievement, took teachers’ expectation pressure and graduation pressure from school as variables and found that these pressures would cause some students to show excessive tension and anxiety during study, which would affect their academic performance and reduce their academic achievement. Bai (2013) found in his research on factors influencing college students’ learning gains that good use of school resources, supportive campus environment, more social interactions and academic activities would encourage students to invest more time and energy in their personal learning, thus promoting students to obtain good learning results. In addition, curriculum of colleges and universities, students’ professional identity, educational function of campus activities, humanistic care of schools and teachers, and learning enthusiasm of classmates also have a significant effect on the improvement of students’ academic achievement.

Most of the factors influencing academic performance were attributed to individual quality and school education, until Coleman et al. (1966) pointed out that school conditions did not have a significant effect on academic performance, but it was the socioeconomic status level of students’ families that significantly affected the chance of academic success. The conclusion of Coleman’s report greatly impacts the traditional concept of school education, and reminds people that the research on the factors influencing academic achievement should not be limited to the individual and school level. Chinese scholars Liu and Lu (2009) and Li (2013) have proved that there is a direct causal relationship between the educational background of parents and the annual income level of the family and their children’s academic performance. The higher the economic status index, the more parents are likely to invest in their children’s education and the more likely their children are to achieve academic excellence. In addition, parents’ professional status may also affect their children’s academic achievement. Fang and Feng (2008) found that the children of managers have the best academic performance, followed by the children of professional and technical personnel, followed by the children of migrant workers, and the children of those who have no fixed occupation have the worst academic performance.

It can be seen that personal factors, school environment and family environment will affect students’ academic achievement to varying degrees, and they jointly affect students’ learning motivation, learning engagement and learning satisfaction. Among them, individual factors and school factors will be connected with family factors to varying degrees to affect students’ academic achievement. Therefore, this paper will mainly study the influence of family capital on students’ academic achievement.

## 3. Theoretical perspective

Group differences in the quality of higher education are a hot issue drawing constant attention in the academics. Currently, scholars mainly analyze the ascribed factors such as family capital as they analyze the influencing factors of academic achievement differences but there are two major effects of family capital on academic achievement: one is the inhibiting effect and the other is the incentive effect (Tan et al., 2018).

### 3.1. The impact of inhibiting effect on the academic achievement

Following the Coleman’s report (Coleman et al., 1966), many scholars have focused on the impact of family capital on students’ academic achievement at different stages. Most studies have shown that students’ academic achievement and family economic level are closely related, that is to say, the higher socioeconomic status the parents are, the higher academic achievement level their children will achieve. For example, Frank McCourt studied the influence of family revenue on the achievement of American high school students. He found that the difference of family revenue will lead to the inequality of educational opportunities and the difference of students’ achievement. In China, with the data from 20 higher institutes of Jiangsu Province, Gao et al. (2011) also found that “family economic status has a significant positive effect on college students’ academic performance during school, and children from high-income families have better academic performance than children from low-and-middle income families.”

The “effective maintenance of inequality” theory suggests that the expanding size of education does not affect the gap in educational quality in spite of the decreasing inequality in access to education formally because the internal disparities in educational quality are still evident. Families in the advantaged class will seek to ensure their children’s education, and they will use their advantages to obtain better educational resources for their children to further maintain their class status. College students from advantaged families with highly educated parents still have their own advantages when they enter college compared to their childhood, and they are more confident and outperform students from poorer areas in terms of academic performance and ability development.

### 3.2. The impact of incentive effect on academic achievement

With the constant strengthening of China’s investment in education and the continuous adjustment of education policies, China’s higher education has been shifting from meritocratic to universal education, the education inequality in form is gradually diminishing, and many students from poor families have more opportunities to enter universities for higher education with the help of favorable policies. Despite many shortcomings, those children from underclass such as workers and farmers are relying on their own efforts to break the monopoly of the upper class on higher elite education. They are willing to pay more for their studies to convert their cultural capital into economic capital to the greatest extent possible, and to put in more effort to get higher income in the future.

While children from advantaged families are using economic resources to “directly” exclude and cultural resources to “invisibly” exclude students from poor families and widen the gap between classes, children from disadvantaged families are striving to bridge the gap as early as they entered the college (Tan et al., 2018). The resilience theory suggests that students from families with low economic and cultural capital but high levels of resilience have the ability to achieve high levels of academic achievement through the investment of non-material resources in family capital that prioritizes education and the stimulation of their own potential.

In addition, with the continuous development of economy, more and more parents from the disadvantaged class are aware of the importance of literacy. At present, education is still one of the most effective ways to achieve class mobility. Studies have found that, in contrast to the argument that “Rags to riches stories are rare,” families of the classes of worker and peasant show higher educational expectations and are willing to invest more in education to bridge the innate gap, motivating their children to rely on education to achieve class leapfrogging. For example, William H, together with other researchers, have found that educational expectations have a significant impact on students’ academic achievement independent of family socioeconomic status and intelligence. Wang and Shi (2014), based on the 2010 Shanghai Household Living Conditions Survey, found that advantaged families have higher educational expectations for their children and their children also have the same expectations for themselves, which are ultimately beneficial to their children’s higher education attainment.

In conclusion, if the inhibitory effect is in dominant position, then there will be a gap between children from advantaged and disadvantaged families. Therefore, many current education preferential policies have achieved equity in terms of goals and structures, but there still have inequity in efficiency, and the advantaged class will still predominate the main educational resources. If the incentive effect dominates, then many children from poor families will still work hard to change their academic achievement status without the impact of family deficit. In this way, both the equity of the goals and structures and equal efficiency are met. However, there is no definite conclusion in the academic field as to which of the two effects of family capital dominates among the factors influencing academic achievement of first-generation college students. Therefore, the hypothesizes are proposed as follows.

*H1*: Family economic capital has a significant positive effect on the academic achievement of first-generation college students.

*H2*: Family cultural capital has a significant positive effect on the academic achievement of first-generation college students.

*H3*: Family social capital has a significant positive effect on the academic achievement of first-generation college students.

Academic achievement could be divided into three dimensions: academic performance, ability development and self-concept (Jia et al., 2020). Thus, in this study, H1 is subdivided into H1a, H1b and H1c; H2 is subdivided into H2a, H2b and H2c; H3 is subdivided into H3a, H3b and H3c. H1a, H1b, H1c, H2a, H2b, H2c, H3a, H3b and H3c are as follows.

*H1a*: FFamily economic capital has a significant positive effect on the ability development of first-generation college students.

*H1b*: Family economic capital has a significant positive effect on the academic performance of first-generation college students.

*H1c*: Family economic capital has a significant positive effect on the self-concept of first-generation college students.

*H2a*: Family cultural capital has a significant positive effect on the ability development of first-generation college students.

*H2b*: Family cultural capital has a significant positive effect on the academic performance of first-generation college students.

*H2c*: Family cultural capital has a significant positive effect on the self-concept of first-generation college students.

*H3a*: Family social capital has a significant positive effect on the ability development of first-generation college students.

*H3b*: Family social capital has a significant positive effect on the academic performance of first-generation college students.

*H3c*: Family social capital has a significant positive effect on the self-concept of first-generation college students.

## 4. Data sources and analysis

### 4.1. Data sources

The data of this study come from questionnaires (see Supplementary Table S1 for details). The questionnaire is based on the CFPS (China Family Panel Studies) database and tailored to the specific circumstances. The CFPS is a nationally representative, biennial comprehensive social survey project initiated by Institute of Social Science Survey, Peking University. Its objective is to document changes in various aspects of Chinese society, economy, population, education, and so on. Prior to the official survey, a small-scale pre-survey was conducted among university students from five universities in Liaoning Province (a total of 330 questionnaires were distributed). The purpose of this pre-survey was to test the rationality of questionnaire item design, completion time, and other factors. Based on the results of the pre-survey, adjustments were made to the wording and content of the survey questionnaire. Additionally, the pre-survey samples were not included in the valid samples for the official survey.

The formal questionnaire takes the method of hierarchical clustering sampling. A total of 2,200 questionnaires were distributed in the formal survey, and 2031 of them filled by the students from five universities in Liaoning Province, and were collected in the end. Of them, valid questionnaires reached to 1,524 after screening 507 questionnaires of non-first-generation college students. According to the statistical results (see Table 1), it can be seen that among the sampled students, there were 363 males and 1,161 females. Among the samples, there were 1,152 students from rural areas, townships, and county-level areas, accounting for 75.6% of the total; 1,086 students were non-only children, accounting for 71.30%. In terms of academic year, there were 604 freshmen, accounting for 39.6%; 321 sophomores, accounting for 21.1%; 372 juniors, accounting for 24.4%; and 227 seniors, accounting for 14.9%. The majority of students in the sample came from regular universities, with majors mainly concentrated in fields such as economics, management, education, law, humanities, history, and philosophy. There were also students from other types of majors.

**Table 1:** Description of sample characteristics.

Variables	Sample classification	Frequency (persons)	Percentage (%)
Gender	Male	363	23.8
	Female	1,161	76.2
Major type	Arts, history, and philosophy	316	20.7
	Science and engineering	201	13.2
	Economics, management, education, and law	857	56.2
	Agriculture, military, and medicine	49	3.2
	Arts and sports	101	6.6
Grade	Freshman	604	39.6
	Sophomore	321	21.1
	Junior	372	24.4
	Senior	227	14.9
Only child or not	Only child	438	28.7
	Non-only child	1,086	71.3
Hometown	Provincial capital or direct-controlled municipality	123	8.1
	Prefecture-level city	249	16.3
	County-level city or county town	438	28.7
	Township	120	7.9
	Rural area	594	39

This study explores whether family capital significantly has a significant impact on academic achievement among first-generation college students? How does family capital affect academic achievement? To answer these questions, this study will use structural equation modeling to conduct an empirical study while verifying the hypothesis of the problems. This study will use AMOS 24.0 software to construct a structural equation model for empirical research. Firstly, the fit between the model and the sample will be tested, and then the specific effects between family capital and the various dimensions of academic achievement in first-generation college students will be analyzed. Finally, the research hypotheses will be validated.

### 4.2. Variable measurement

The dependent variable of this study is academic achievement, including academic performance, ability development and self-concept. Independent variable is family capital, which includes economic capital, cultural capital and social capital (see Supplementary Table S1 for details). The meanings of variables are as follows.

#### 4.2.1. Academic achievement

The purpose of measuring academic achievement is to evaluate the extent to which students have reached the expected learning goals. It not only focuses on students’ learning outcomes but also considers their comprehensive development in various aspects during the learning process. This study mainly draws on the definition of Jia et al. (2020) as the reference, defining the academic achievement as the academic performance gained by the students (professional achievement, professional skills, and overall performance ranking, etc.), ability development (problem solving, communication and negotiation, organizational leadership, and effective cooperation), and self-concept (self-perception, learning satisfaction, and achievement attribution, etc.). The reason for referencing this definition is twofold. Firstly, their work clarifies the concept of academic achievement with three dimensions, which helps operationalize the concept in this study. Secondly, their study shares similarities with this research as it also examines a relatively disadvantaged group of college students, demonstrating strong intergenerational transmission. For the measurement of each dimension of academic achievement, the Likert Scale is used in the study with five levels from low to high, each assigned a score of 1–5. The Cronbach’s α of this scale is 0.831. The CR values of ability development, academic performance and self-concept are 0.929, 0.940 and 0.946 respectively. The AVE values of ability development, academic performance and self-concept are 0.766, 0.798 and 0.853 respectively.

#### 4.2.2. Family capital

Primarily based on Bourdieu (1986), he points out in *The Forms of Capital* that the three basic forms of capital are economic capital, cultural capital, and social capital.

In the aspect of economic capital, this study follows Bourdieu’s view that economic capital is a type of capital that can be directly converted into money, exists mainly in material form, and is institutionalized in the form of property, such as possessions, houses, number of cars, and other consumption items, and is a type of explicit capital. This paper also points out in the literature review that family economic capital is embodied in terms of annual family income and the status of inherent assets, which reflects the level of parents’ investment in their children’s education and the material support for their children to enjoy educational resources. Meanwhile, this paper refers to the research of Liu and Gao (2011) and Zhang and Lin (2022), economic capital in this study refers to the annual income of the family, parents’ occupational status, and investment in children’s education. Therefore, five questions are designed, which are: “the total annual income of your family” “the per capita monthly income of your family is approximately” “your family’s contribution to your university expenses (tuition and living expenses)” “your father’s occupation” “your mother’s occupation.”

In the aspect of cultural capital, family cultural capital refers to the possession of family cultural resources, including institutionalized family cultural capital, specific family cultural capital and objective family cultural capital (Bourdieu, 1986). Objective cultural capital is embodied in the form of cultural goods, including books, dictionaries, tools and other family possessions. Specific cultural capital is embodied in the temperament tendency internalized in human spirit and body, such as internalized language, skills, tastes, behaviors, knowledge systems, etc. Institutionalized cultural capital is the capital obtained through some system confirmation, such as vocational certificates, academic certificates and award certificates with social value granted by authoritative institutions such as schools and government agencies. Research shows that parents’ education level and family book resources have important influence on the development of students’ reading ability. Scarborough and Dobrich (1994) found that parents’ education level had a positive effect on children’s reading ability. McQuillan and AU (2001) found that household book collections were positively correlated with reading frequency and reading achievement. This article refers to the research of Li and Tan (2019) and Li et al. (2022), and designs four questions for family cultural capital, which are: “your father’s highest education qualification” “your mother’s highest education qualification” “your family book collection” “how often do your family members take you to participate in cultural activities (including viewing exhibitions, visiting museums, etc.).”

In the aspect of social capital, this paper points out that family social capital mainly refers to the parent–child relationship within the family, parents’ educational expectation for their children, and parents’ external social relationship and the utilization degree of parents’ social relations. Marjoribanks and Mboya (2001) also argue that family social capital mainly refers to the relationship between family members, and that for individuals, parents in the family are important subjects of family social capital. In addition, this paper refers to the research of Zheng (2012) and Du and Ding (2014), and designs 4 questions for family social capital, which are: “when encountering matters related to you, your family members will discuss with you in advance” “the number of times your parents communicate with you each month” “your family has a wide circle of friends” “when you need, you can get help from relatives and friends.” Therefore, this study divides family capital into economic capital (annual income of the family, parents’ occupational status, and investment in children’s education), cultural capital (parents’ literacy, book collection at home, and frequency of participation in cultural activities), and social capital (including parents’ interpersonal network, satisfaction with the help provided by relatives and friends, and frequency of communication between parents and children). In each of the questions, five scales were assigned in descending order, with higher scores indicating higher capital stock in that dimension. For the measurement of each dimension of family capital, the Likert Scale is used in the study with five levels from low to high, each assigned a score of 1–5. The Cronbach’s α of this scale is 0.856. The CR values of economic capital, cultural capital and social capital are 0.936, 0.869 and 0.943 respectively. The AVE values of economic capital, cultural capital and social capital are 0.744, 0.624 and 0.805 respectively.

#### 4.2.3. The family capital level and academic achievement level of the sample

As can be seen from Table 2, the average score of the family economic capital dimension is 10.67, and the mode is 7. The average value is higher than the mode, indicating that the overall score of the sample is low. The average score of family cultural capital dimension is 12.05, and the mode is 12. The two are close but the mode is low, indicating that the sample cultural capital scores are at a low level, and it can be preliminarily determined that the cultural capital level of the respondents is below the middle level. The average score of family social capital dimension is 12.93, and the mode is 13. The two are close, indicating that the sample social capital scores are at a medium level, and the social capital level of the respondents can be preliminarily determined to be medium.

**Table 2:** Score table of family capital in each dimension.

	Number	Items	Average	Modal number	Standard deviation
Economic capital	1,524	5	10.67	7	3.76
Cultural capital	1,524	4	12.05	12	1.56
Social capital	1,524	4	12.93	13	2.63

Through descriptive statistics on each dimension of the academic achievement of first-generation college students, such as the mean and standard deviation, kurtosis and skewness, the results are shown in Table 3. The data show that the minimum value of each item of the academic achievement of first-generation college students is 1, and the maximum value is 5. The mean values of items NO.25, NO.27, NO.28, NO.38 are less than 3, and the mean values of other items are slightly higher than 3. Therefore, the academic level of first-generation college students is relatively low and is below the medium level.

**Table 3:** The statistics of the academic achievement scale.

Item number	Minimum	Maximum	Average	Standard deviation	Variance	Skewness	Kurtosis
25	1	5	2.54	1.110	1.232	0.415	−0.459
26	1	5	3.51	1.252	1.567	−0.439	−0.805
27	1	5	2.24	1.050	1.102	0.399	−0.627
28	1	5	2.13	0.954	0.910	0.365	−0.566
30	1	5	3.22	0.798	0.637	−0.133	0.788
31	1	5	3.52	0.772	0.596	−0.223	0.751
32	1	5	3.29	0.778	0.606	−0.062	0.776
33	1	5	3.46	0.771	0.595	−0.201	0.460
36	1	5	3.29	0.724	0.524	0.035	0.750
37	1	5	3.32	0.768	0.590	0.069	0.412
38	1	5	2.84	0.800	0.640	0.369	0.782

### 4.3. Empirical findings

#### 4.3.1. Correlation test between the dimensions of each variable

In this study, SPSS 23.0 was used to control for the above factors, and a partial correlated two-tail test was conducted on academic achievement and its influencing factors.

It can be seen from Table 4 that economic capital is significantly correlated with cultural capital, and the correlation coefficient *r* = 0.056, *p* < 0.05, a weak correlation. Economic capital shows a significantly positive correlation with social capital, and the correlation coefficient *r* = 0.369, *p* < 0.01, a weak correlation; a significantly positive correlation with ability development, the correlation coefficient *r* = 0.266, *p* < 0.01, which is weak correlation; a significantly positive correlation with academic performance, correlation coefficient *r* = 0.069, *p* < 0.01, which is weak correlation; a significantly positive correlation with self-concept, and the correlation coefficient *r* = 0.178, *p* < 0.01, a weak correlation. There is a significantly positive correlation between ability development and social capital, and the correlation coefficient *r* = 0.407, *p* < 0.01, which is moderate correlation. There is a significantly positive correlation between self-concept and academic achievement, *r* = 0.531, *p* < 0.01, a moderate correlation.

**Table 4:** Correlation matrix.

	Economic capital	Cultural capital	Social capital	Ability development	Academic achievement	Self-concept
Economic capital	1	0.056*	0.369**	0.266**	0.069**	0.178**
Cultural capital	0.056*	1	0.210**	0.136**	0.056*	0.145**
Social capital	0.369**	0.210**	1	0.407**	0.041	0.344**
Ability development	0.266**	0.136**	0.407**	1	0.056*	0.531**
Academic achievement	0.069**	0.056*	0.041	0.056*	1	−0.052*
Self-concept	0.178**	0.145**	0.344**	0.531**	−0.052*	1

*Significant relation at level 0.05.

**Significant relation at level 0.01.

In conclusion, family capital is significantly and positively correlated with the academic achievement of first-generation college students, and the three dimensions of family capital (economic capital, cultural capital, and social capital) are also significantly and positively correlated with the three dimensions of academic achievement (ability development, academic achievement, and self-concept), that is to say, the higher the stock of family capital is, the higher academic achievement the first-generation college students gains.

#### 4.3.2. Construction and measurement of SEM model of influencing factors on academic achievement of first-generation college students

Based on the research results of the correlation analysis of variables in economic capital, cultural capital, social capital and variables in academic achievement, this study uses AMOS.24 software to construct a structural equation model to further analyze the influencing factors between each capital and academic achievement. As a result, the research hypothesis of this study can be verified.

**Figure 1:**
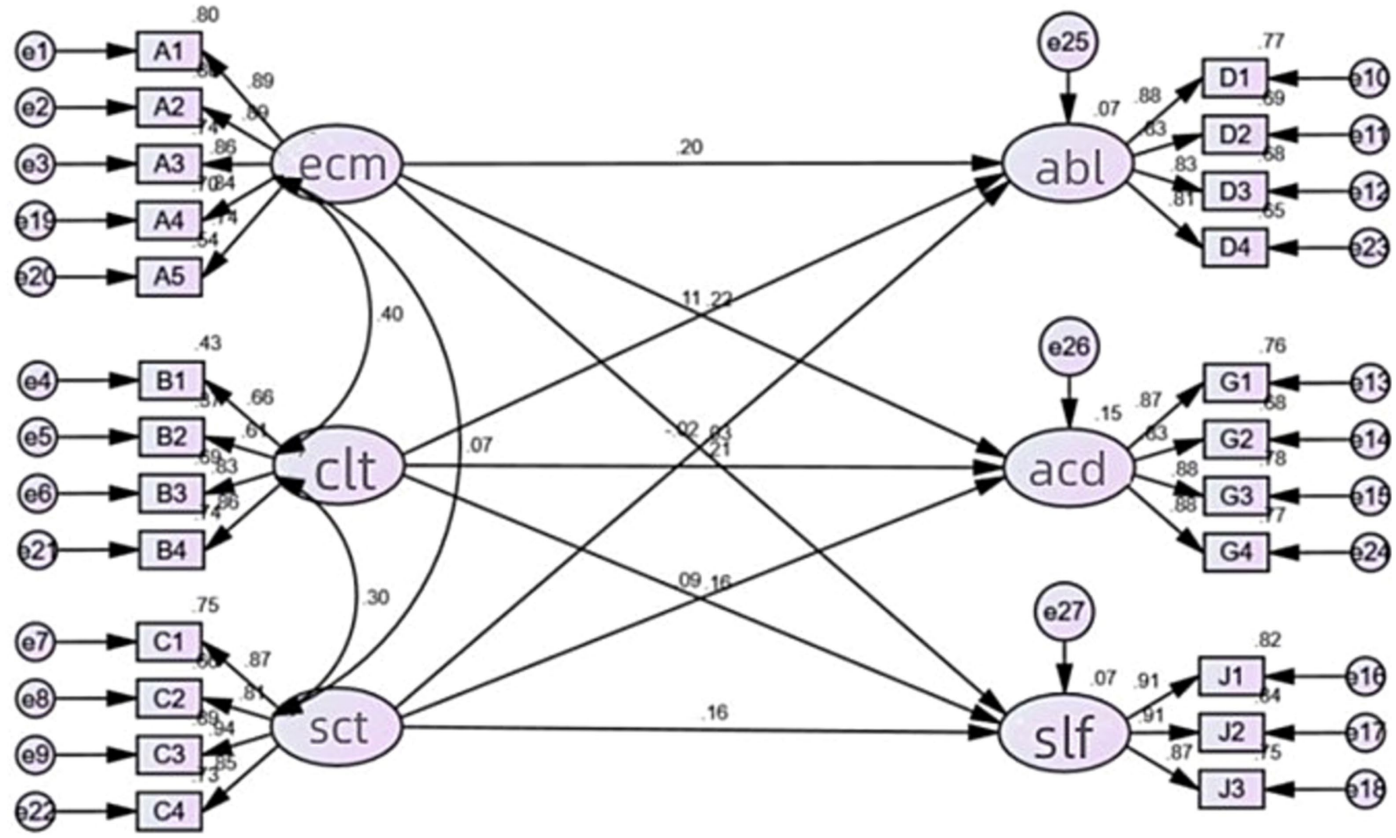
Initial model. This figure shows the initial structural equation model of family capital and academic achievement of first-generation college students. ECM means economic capital, CLT means cultural capital, SCT means social capital, ABL means ability development, ACD means academic performance, SLF means self-concept.

In this study, the three-dimension family capital was taken as the external potential variable and the three-dimension academic achievement was taken as the internal potential variable. Error terms were added to each measurement dimension and internal potential variable to construct the structural equation model of family capital and academic achievement of first-generation college students (see Figure 1), and the mechanism of the two was explored.

**Figure 2:**
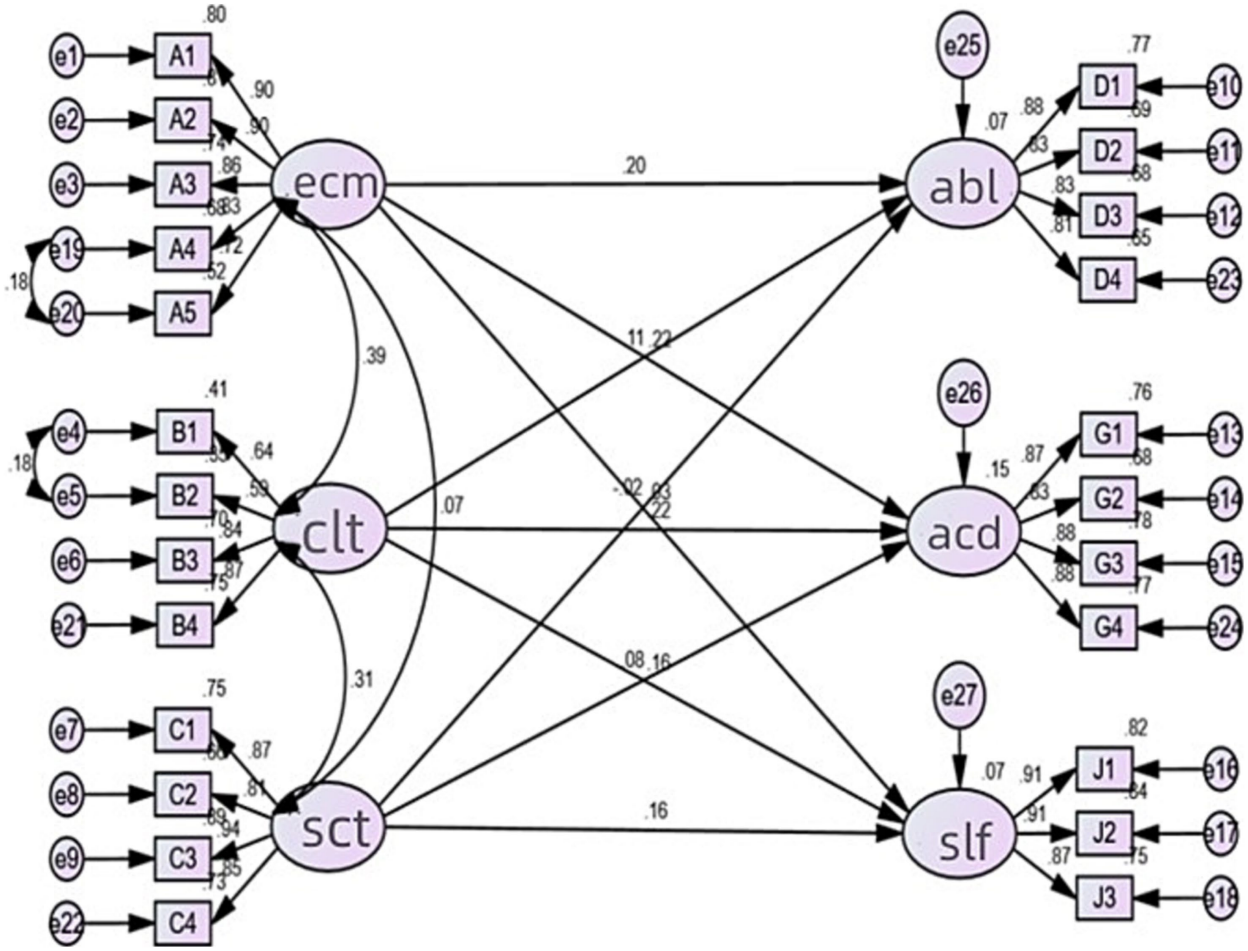
Modified structural equation model. This figure shows the initial structural equation model of family capital and academic achievement of first-generation college students.

Test results of model fit degree of initial model show that χ^2^/df = 5.250, which is not satisfactory (Table 5). MI correction can be performed according to the model correction index provided by AMOS output. The covariation relationship added in the correction model reflects the same high-order factor and satisfies the SEM model, and only one covariant relationship can be added at a time. The revised model is shown in Figure 2.

**Table 5:** Test results of model fit degree of initial model.

Index	χ^2^/*df*	NFI	RMSEA	GFI	AGFI	RFI	TLI	CN	*P*
Numerical value	5.250	0.953	0.053	0.935	0.919	0.946	0.956	332	0.000
Criterion	<5	>0.90	<0.08	>0.90	>0.90	>0.90	>0.90	>200	<0.01

The modified model is tested for fitness, and all fitness indicators meet the test standards and achieve the ideal fitness. The test results are shown in Table 6.

**Table 6:** Test results of fit degree of modified model.

Index	χ^2^/*df*	NFI	RMSEA	GFI	AGFI	RFI	TLI	CN	*P*
Numerical value	4.959	0.956	0.051	0.940	0.924	0.934	0.959	377	0.000
Criterion	<5	>0.90	<0.08	>0.90	>0.90	>0.90	>0.90	>200	<0.01

It can be seen from Table 6 that for the revised model, *χ*^2^*/df* = 4.959<5, NFI = 0.956 > 0.90, RMSEA = 0.051 < 0.08, GFI = 0.940 > 0.90, AGFI = 0.924 > 0.90, RFI = 0.934 > 0.90, TLI = 0.959 > 0.90, CN = 377 > 200, P = 0.000 < 0.01, indicating that the modified model has good fit with the sample data. The Maximum Likelihood method is used to estimate the standardized and unstandardized regression coefficients and their significance. The results are shown in Table 7.

**Table 7:** Regression coefficients.

	Unstandardized regression coefficient	Standardized regression coefficient	S.E.	C.R.	*P*
Ability development <−-- economic capital	0.171	0.198	0.026	6.538	***
Academic performance <−-- economic capital	0.224	0.220	0.029	7.650	***
Self-concept <−-- economic capital	0.033	0.034	0.029	1.152	0.249
Ability development <−-- cultural capital	0.141	0.114	0.041	3.424	***
Academic performance <−-- cultural capital	0.315	0.217	0.047	6.716	***
Self-concept <−-- cultural capital	0.221	0.160	0.046	4.833	***
Ability development <−-- social capital	−0.018	−0.020	0.026	−0.701	0.483
Academic performance <−-- social capital	0.089	0.084	0.029	3.058	0.002
Self-concept <−-- social capital	0.157	0.156	0.029	5.472	***

*** indicates *P*<0.001.

From the results in Table 7, it can be seen that for the path of “Self-concept <−-- economic capital” and “Ability development <−-- social capital,” P is bigger than 0.05, which shows that these paths are not significant. For other paths, the C.R values are greater than 1.96, which means that these paths are significant. Thus, H1a, H1b, H2a, H2b, H2c, H3b, and H3c are all confirmed. Through the standardized regression coefficients, it can be seen that for the family economic capital, it has greater impact on academic performance than on ability development. For the family cultural capital, it has the greatest impact on academic performance, followed by self-concept and ability development; for family social capital, it has greater impact on self-concept than on academic performance.

#### 4.3.3. Research hypothesis verification results

Above all, the model of family capital and academic achievement has achieved an ideal fit. The impact path and degree of family capital on academic achievement, the impact path and degree of family economic capital, family social capital, and family cultural capital on the academic achievement of first-generation college students (ability development, academic performance, self-concept) are all in the above. The model verification process is displayed, and all the hypotheses are verified in the model running results. The specific research hypothesis testing results are shown in Table 8.

**Table 8:** Summary of test results of research hypothesis.

Serial number	Hypothetical description	Test result
H1a	The influence of family economic capital on ability development	Significant
H1b	The influence of family economic capital on academic performance	Significant
H1c	The influence of family economic capital on self-concept	Insignificant
H2a	The influence of family cultural capital on ability development	Significant
H2b	The influence of family cultural capital on academic performance	Significant
H2c	The influence of family cultural capital on self-concept	Significant
H3a	The influence of family social capital on ability development	Insignificant
H3b	The influence of family social capital on academic performance	Significant
H3c	The influence of family social capital on self-concept	Significant

## 5. Conclusion

The quality of higher education is a hot issue that the academic community is more concerned about, and it is also an educational problem related to personnel training and sustainable social development. First-generation college students, as the main representative of the student group, have many influencing factors that cause the difference in academic achievement.

Based on the ascribed factor of family, many scholars have analyzed the differences in the impact of economic capital, cultural capital and social capital in the family on the academic achievement of college students. However, it is not clear whether these factors have a direct and significant impact on the academic achievement of first-generation college students and how they act on the academic achievement. This study explores the influence path of family capital factors on academic achievement through structural equation model, and the results show that family cultural capital has a direct positive impact on the academic achievement of first-generation college students. Among them, economic capital has a greater impact on the academic performance and ability development of the first-generation college students, but has no significant positive impact on self-concept. Social capital has a major impact on the academic performance and self-concept of the first-generation college students, but has no significant effect on ability development. Generally speaking, the higher the educational level of parents in the family, the more their children are influenced by their parents, and their academic achievements will be relatively prominent. In addition, the improvement of the economic and social status in the family will also provide material support for the children’s educational investment, and the parents’ ability levels are also passed on from generation to generation. At the same time, the social network of parents in the family and the support provided by relatives and friends will also indirectly affect the children. Good interpersonal communication will subtly affect the values and attitudes of children and help children form correct self-cognition.

In conclusion, the “inhibitory effect” of family capital in higher education is still dominant, and the overall impact on academic achievement is significant.

Indeed, relevant research shows that the family economic disadvantaged college students have the characteristics of “low starting point, rapid development,” their family economic background plays the role of incentives rather than inhibition and helps them catch up with other college students in ability (Tan et al., 2018). But this “incentive effect” only exists in some elite institutions, where students rely on high-quality academic performance to obtain high-quality secondary school admission opportunities and resources to make up for the lack of cultural capital of their families, and through self-adjustment and efforts to achieve academic resilience (Zhang and Zhu, 2018).

## 6. Recommendations

As an innate factor, family capital has an important impact on students at different stages of school age. In the stage of entering higher education, in addition to structural factors such as family economic level, parents’ educational level, family cultural atmosphere and parents’ social interpersonal relationship, school capital should also be noticed. The school factor has been absent for a long time in the research on social inequality in our country. The perspective of Western “conflict theory” often regards schools as “filters” and “agents of the upper middle class” (Ju and Liu, 2022). The study of schools in the conflict theory is based on Weber’s theory of cultural conflict, which holds that education is controlled by the dominant class and is the transmission of the identity culture of the dominant class. Afterwards, it continued to develop, and the most representative theory in the conflict theory is Bourdieu’s cultural capital theory. Bourdieu’s cultural capital theory emphasizes that children bring the behavior habits soaked in the family to school, which leads to different preferences of the school. The habitus of the upper class is favored and rewarded by the school, which ultimately promotes the educational success of its children, while the educational results of the lower-class children are the opposite (Liu, 2013). In particular, first of all, the conflict theory emphasizes the screening function of education, and holds that the screening mechanism in education implies the class preference of school education, and that the competition of different classes for education is accomplished in the process of education screening, the criterion for screening is the behavioral characteristics of the dominant class. Secondly, conflict theory holds that the stratification of education is the result of class struggle, which is mainly affected by family background. Finally, conflict theory holds that for individuals, rule internalization leads to the results of screening, and rule internalization of different classes leads to different educational achievements (Liu and Hu, 2012). However, this perspective actually ignores the realistic gap between western countries and Chinese society, and ignores the positive role of schools. In the field of higher education, how to weaken the impact of family capital gap on academic achievement, give full play to the positive role of school capital, and achieve effective class mobility. Based on this, the following implications are drawn:

### 6.1. Recommendation 1. Pay close attention to the internal actions of students in the field of higher education

In examining academic achievement, previous studies have ignored the group differences of students, limited to the acquisition of educational resources, and seldom considered the real acquisition of resources and the degree of mastery of utilization. Students are slightly different in micro-levels such as personality characteristics, habitual acquisition, and social cognition. Structural duality (Giddens, 2016) holds that, on the one hand, society itself has structures, which constrains people. On the other hand, people should not only recognize the original social structure, but also adjust behavioral rules and social institutions. Similarly, as the main body of higher education, we should not only learn to use educational resources, but also strengthen the interaction between resources and rules. In other words, while using educational resources, we must strive to change the rules according to their own needs, so as to meet new educational needs. In addition, Vroom’s “expectation theory” believes that the greater a person estimates the value of the goal, the greater the probability of the estimated goals will be achieved, and the greater the motivation for them. Therefore, in the field of higher education, students themselves should strengthen their expectations of achievement development and self-fulfillment, so as to inspire them to make more efforts in practical actions, give full play to their subjective initiative, and embed their practical actions into objective. At the same time, in the school environment, for students, the self-contained factors are constantly strengthened, and the impact of the deficit caused by the ascribed factors is reduced, which is conducive to moving toward higher-level goals. Finally, the main body of students should make full use of school capital to make up for the lack of family capital. The theory of resilience explores the inherent advantages of first-generation college students from a positive psychological perspective. This theory explains that individuals show stronger adaptability in adversity. When they find that their objective conditions are insufficient, they will be willing to put in more efforts and actions to make up for the innate gap. After first-generation college students enter the university stage, on the one hand, they must give full play to their own subjective initiative, and on the other hand, they must learn to transform the educational and cultural resources of the school into their own cultural capital, and eliminate the conflict between themselves and non-first-generation college students through effective interaction with the school environment. At present, faculty and staff in Chinese universities generally belong to first-generation college students. They have similar family background and study experiences with first-generation college students. They can play a role of example and demonstration in daily contact and teaching process, so as to strengthen effective teacher-student interaction and stimulate students’ academic enthusiasm.

In addition, related research shows that when the first-generation group of students cannot obtain a better educational experience from their parents, they will turn this sustenance to the teachers of the school, thereby improving the quality of teacher-student interaction and benefiting students’ educational gains.

### 6.2. Recommendation 2. Strengthen the interaction between different fields

At present, when studying the academic achievements of first-generation college students, scholars mostly focus on the representation of different fields and the internal operation mechanism, ignoring the mutual linkage and influence between different fields (Shen, 2021). First, in the family field, although the parents of first-generation college students have not received higher education and lack of experiences in education, they should give strong support in education investment, give their children high educational expectations so as to bring honor to the family through education. Relevant studies have shown that there are still a large number of families in rural China that cannot afford higher education costs, but material support from family relatives still plays an important role in their children’s education. Therefore, the linkage between small family and large family of China’s first-generation college students may still make up for the lack of capital. Second, for the field of basic education, the “fault phenomenon” between basic education and higher education still exists (Ding and Li, 2022). Education is a continuous and interconnected process, and educational achievements cannot be achieved overnight. The accumulation of education in the previous stage is of great significance to the subsequent stage. Relevant studies have shown that the learning habits and quality formation in high school have an important impact on the adaptation to college. Therefore, in the whole process of education, we should do a good job of connecting tasks at each stage, and strengthen the effective interaction between higher education and basic education. For the difficulties and deficiencies that are about to enter the university stage, basic education should make preparations in advance, and especially for children from lower-level families, more attention should be paid.

### 6.3. Recommendation 3. Focus on education fairness and implement humanistic care for disadvantaged groups

At present, with the continuous development of China’s economy, China is paying more and more attention to the issue of education fairness. The introduction of preferential policies for education has helped those families in difficulty, so that children from remote areas can go to school. However, in recent years, one-size-fits-all assistance projects for various vulnerable groups have not been able to effectively meet the complex needs of first-generation college students, and the “simple and rude” funding policies of major universities have not achieved substantial results. When dealing with the educational fairness of first-generation college students, China’s colleges and universities can also learn from the relevant experience of foreign countries, such as the intervention project experiences of the University of Maryland in the United States. Chinese universities can also collect students’ family situation in advance, create different learning and living environments according to the number of first-generation college students in the university, formulate different dormitory allocation policies and arrange counselors with similar family backgrounds, give effective care for life and learning, and do auxiliary work for better adaptation to university life. In addition, schools should also formulate different teaching modes according to the predicaments of the first-generation college students, such as reducing the direct teaching, encouraging students to discover problems independently, and strengthening effective interaction with teachers in the process of guidance, so as to prevent the first-generation college students from entering new unconscious conflict because of inappropriate campus environment. In addition, when higher education alleviates the impact of disadvantaged family, the support of teachers and peers is also an important driving force for the development of first-generation college students. Teachers should not only play the role of good teachers, but also life mentors on the way of students’ development, to increase students’ self-confidence, explore students’ potential, and improve students’ cognitive level. School can also strengthen the communication between students by holding various cultural activities, so that students can find suitable companions for them, and can also share the inspirational stories of the senior students to stimulate the fighting spirit of the junior students, narrow the educational gap, and improve their academic achievements.

## Data availability statement

The raw data supporting the conclusions of this article will be made available by the authors, without undue reservation.

## Ethics statement

The studies involving human participants were reviewed and approved by the Institute of Higher Education, Jilin University. The ethics committee waived the requirement of written informed consent for participation.

## Author contributions

ZW has made substantial contributions to the conception and design of the work, designed the theoretical framework, and research methods of the manuscript, and contributed to the revision of the manuscript, the acquisition, analysis, and interpretation of data for the work. YZ has made great contributions to the design of the research framework, wrote the abstract, literature review, introduction, theoretical perspective, and conclusion, drafted, and wrote the interpretation of data of the manuscript. ZR wrote the recommendation section, helped to perform the statistical analysis, and wrote the data sources and analysis. All authors have collected the data, helped write the first draft of the manuscript, revised the manuscript several times, and approved the submitted version.

## Funding

This work supported by the Research Project for Humanities and Social Sciences, Ministry of Education (21YJA880053) and Research Project of Jilin Provincial Social Science Fund (2022B143).

## Conflict of interest

The authors declare that the research was conducted in the absence of any commercial or financial relationships that could be construed as a potential conflict of interest.

## Publisher’s note

All claims expressed in this article are solely those of the authors and do not necessarily represent those of their affiliated organizations, or those of the publisher, the editors and the reviewers. Any product that may be evaluated in this article, or claim that may be made by its manufacturer, is not guaranteed or endorsed by the publisher.
