# Supplementary material for: Research on the influence of family capital on academic achievement of first-generation college students in China
Source: Front Psychol. 2023 Aug 24;14:1174345. doi: 10.3389/fpsyg.2023.1174345 (PMC10484505; doi:10.3389/fpsyg.2023.1174345)
Supplement: Supplementary file 1 [file Table_1.DOCX]

Supplementary Material

**Research on the Influence of Family Capital on Academic Achievement of First-Generation College Students in China**

Ying Zhao, Zengyuan Ren, Zhi Wang*

*Correspondence: Zhi Wang: 1215762513@qq.com

Table S1.

| No. | Items | Dimensions |
| --- | --- | --- |
| 1 | **Your gender:**  (A. Male B. Female) | Basic Personal Information |
| 2 | **What is your current major?**  (A. Humanities and Social Sciences B. Science and Engineering C. Economics, Management, Education, and Law D. Agriculture, Military, and Medicine E. Arts and Sports) | Basic Personal Information |
| 3 | **Your grade:**  (A. Freshman B. Sophomore C. Junior D. Senior) | Basic Personal Information |
| 4 | **Are you an only child?**  (A.Yes B. No) | Basic Personal Information |
| 5 | **Are you the first-generation college student in your family (this research refers to full-time general college undergraduates whose parents have not received a college degree or above?**  (A. Yes B. No) | Basic Personal Information |
| 6 | **The place of your household registration at the time of the college entrance exam belongs to:**  (A. Provincial capital city or municipality directly under the Central Government B. Prefecture-level city C. County-level city or county seat  D. Township E. Rural area) | Basic Personal Information |
| 7 | **Your school category:**  (A. Higher Vocational College B. General undergraduate colleges C. "Double first-class" construction colleges and universities) | Basic Personal Information |
| 8 | **The total annual income of your family:**  (A. Below 36,000 yuan B. 36,000-84,000 yuan C. 84,000—180,000 yuan D.180,000—360,000 yuan E.360,000 or more) | Economic Capital |
| 9 | **The per capita monthly income of your family is approximately:**  (A. Below 1000 yuanB.1000-3000 yuan C. 3000-5000 yuan D.5000-7000 yuan E. More than 7000 yuan) | Economic Capital |
| 11 | **Your family’s contribution to your university expenses:**  (A. Very high pressure B. High pressure C. General D. Low pressure E. No pressure) | Economic Capital |
| 21 | **Your father’s occupation:**  (A. Urban and rural unemployed, unemployed, semi-unemployed people or agricultural laborers B. Workers or business service personnel C. Individual industrial and commercial households or general office workers D. Professional technicians or private business owners E. Senior managers or government leaders) | Economic Capital |
| 22 | **Your mother’s occupation:**  (A. Urban and rural unemployed, unemployed, semi-unemployed people or agricultural laborers B. Workers or business service personnel C. Individual industrial and commercial households or general office workers D. Professional technicians or private business owners E. Senior managers or government leaders) | Economic Capital |
| 12 | **Your father’s highest education qualification:**  (A. Master degree or above B. University <including junior colleges and higher vocational schools> C. High school <including technical secondary school, vocational high school> D. Junior high school E. Primary school or below) | Cultural Capital |
| 13 | **Your mother’s highest education qualification:**  (A. Master degree or above B. University <including junior colleges and higher vocational schools> C. High school <including technical secondary school, vocational high school> D. Junior high school E. Primary school or below) | Cultural Capital |
| 14 | **Your family book collection:**  (A. None B. Few C. General D. Many E. A lot) | Cultural Capital |
| 15 | **How often do your family members take you to participate in cultural activities (including viewing exhibitions, visiting museums, etc.)?**  (A. Never B. Rarely C. Sometimes D. Often E. Very often) | Cultural Capital |
| 16 | **When encountering matters related to you, your family members will discuss with you in advance?**  (A. Completely disagree B. Disagree C. General D. Agree E. Totally agree) | Social Capital |
| 18 | **The number of times your parents communicate with you each month:**  (A. Never B. Rarely C. Sometimes D. Often E. Very often ) | Social Capital |
| 20 | **Your family has a wide circle of friends.**  (A. Completely disagree B. Disagree C. General D. Agree E. Totally agree) | Social Capital |
| 24 | **When you need, you can get help from relatives and friends.**  (A. Completely disagree B. Disagree C. General D. Agree E. Totally agree) | Social Capital |
| 25 | **Your performance rank in the class:**  (A. Top 10% (including) B. 10% - 30% (including) C. 30% - 50% (including) D. 50% - 70% (including) E. Bottom 30%) | Academic Performance |
| 26 | **The accumulative time you have arranged for studying each week is (in addition to the class time for participating in the class schedule).**  (A. More than 35 hours B. 28-35 <inclusive> hours C.21-28 <inclusive> hours D.14-21 <inclusive> hours E. 14 <inclusive> hours or less) | Academic Performance |
| 27 | **How about the rewards you received during college (such as scholarships, social activists, outstanding student cadres, etc.)?**  (A. Never B. Few C. General D. Many E. A lot) | Academic Performance |
| 28 | **The relevant qualification certificates you have obtained (such as English, computer, professional related, etc.):**  (A. None B. Few C. General D. Many E. A lot) | Academic Performance |
| 30 | **I have organizational leadership skills.**  (A. Completely disagree B. Disagree C. General D. Agree E. Totally agree) | Ability Development |
| 31 | **I have good relationships.**  (A. Completely disagree B. Disagree C. General D. Agree E. Totally agree) | Ability Development |
| 32 | **I have good skills in verbal expression, communication, and conversation.**  (A. Completely disagree B. Disagree C. General D. Agree E. Totally agree) | Ability Development |
| 33 | **I can control my emotions well when facing pressure.**  (A. Completely disagree B. Disagree C. General D. Agree E. Totally agree) | Ability Development |
| 36 | **I can arrange my time reasonably.**  (A. Completely disagree B. Disagree C. General D. Agree E. Totally agree) | Self-Concept |
| 37 | **I have a clear plan for my studies.**  (A. Completely disagree B. Disagree C. General D. Agree E. Totally agree) | Self-Concept |
| 38 | **Satisfaction with self-study.**  (A. Very satisfied B. Fairly satisfied C. Neither satisfied nor dissatisfied D. Fairly dissatisfied E. Very dissatisfied) | Self-Concept |
